# JAK inhibitor tofacitinib alleviates secretory dysfunction and Th17/Treg imbalance in a Sjögren’s disease murine model

**DOI:** 10.1080/07853890.2026.2625552

**Published:** 2026-02-08

**Authors:** Yanjun Lin, Yanjing Ou, Jingjing Su, Jianghan Xu, Chaowei Liu, Kaixun He, Lin Zhou, Dong Wu, Jiang Chen

**Affiliations:** a Fujian Key Laboratory of Oral Diseases, School and Hospital of Stomatology, Fujian Medical University, Fuzhou, China; b Research Center of Dental and Craniofacial Implants, School and Hospital of Stomatology, Fujian Medical University, Fuzhou, China; c Department of Oral Implantology, School and Hospital of Stomatology, Fujian Medical University, Fuzhou, China; d Stomatological Hospital of Xiamen Medical College, Xiamen, Fujian, China

**Keywords:** Sjögren’s disease, transcriptome, tofacitinib, JAK-STAT signalling pathway, Th17/treg balance

## Abstract

**Objectives:**

To examine whether the JAK inhibitor tofacitinib alleviates secretory dysfunction and modulates Th17/Treg balance in a Sjögren’s disease (SjD) murine model.

**Methods:**

Integrated analysis of SjD transcriptome sequencing (GSE159574, GSE247662) identified key signalling pathways, potential therapeutic agents, and immune cell infiltration. NOD/ShiLtj mice were administered with or without tofacitinib. Secretory function and inflammation were assessed via fluorescein ocular surface staining, tear flow rate, histopathology (HE, Masson, Sirius Red), saliva flow rate, immunohistochemistry, immunofluorescence, flow cytometry, and cytokine measurement. Pearson’s linear regression evaluated the correlation between Th17/Treg balance and secretory function.

**Results:**

Bioinformatics analysis showed the JAK-STAT pathway and CD4+ T cells contribute to SjD pathogenesis. Tofacitinib reduced corneal fluorescein staining, increased tear break-up time and secretion, diminished salivary gland lymphocytic inflammation, improved saliva flow rate, and altered phospho-JAK3-STAT1 expression. It also reduced Th17 cell proportion, increased Treg cell proportion in salivary glands and spleens, decreased IL-17, and increased IL-10 and TGF-β in blood. A strong negative correlation existed between secretory function and Th17/Treg balance.

**Conclusions:**

Tofacitinib potently attenuated secretory dysfunction and inflammation in SjD mice, possibly by modulating Th17/Treg balance, suggesting it may be a therapeutic agent for SjD.

## KEY MESSAGES

- The JAK inhibitor tofacitinib may have therapeutic potential in SjD.
- The therapeutic impact of tofacitinib on SjD may be attributable to its capacity to regulate Th17/Treg equilibrium.

## Introduction

Sjögren’s disease (SjD) is a chronic systemic immune-mediated inflammatory disease characterized by focal lymphocytic infiltration of the exocrine glands, particularly the salivary and lacrimal glands [1]. Although most SjD manifests as dry conjunctivitis, xerostomia, and autoantibodies (anti-SSA/Ro and anti-SSB/La antibodies), it can also lead to serious complications, such as pulmonary fibrosis and B-cell lymphoma [2–4]. The current diagnostic criteria for SjD are mainly the International Classification Criteria revised in Europe and the USA in 2002, and the classification criteria developed by the American College of Rheumatology and the European League Against Rheumatism in 2016 [5,6]. The criteria are based on 5 items: anti-SSA antibody positivity, a focal lymphocytic sialadenitis score of ≥1 foci/4 mm^2^, an ocular staining score of ≥5, a Schirmer’s test result of ≤5 mm/5 min, and an unstimulated salivary flow rate of ≤0.1 mL/min [6]. Owing to the complicated pathophysiology of SjD, mainstream treatment aims at immunomodulatory therapies to alleviate sicca complaints and control inflammation.

Th17 and Treg cells differentiate from naive CD4^+^ T cells and ultimately fulfil opposite functions. Th17 cells induce inflammation and autoimmunity, whereas Treg cells inhibit Th17 cell function and maintain immune homeostasis, which is referred to as the Th17/Treg balance [7]. The balance between Th17 and Treg cells may play a critical role in SjD because these subsets are mutually regulated by common and distinct cytokines and can convert to each other under certain inflammatory conditions. Elevated levels of Th17-related cytokines are expressed in the salivary glands of patients with SjD [8]. The quantity of CD4^+^CD25^+^FoxP3^+^ regulatory T cells within minor salivary glands is elevated among individuals diagnosed with SjD, suggesting the migration of regulatory T cells from the bloodstream to the inflamed tissue for anti-inflammation [9]. Consequently, it can be postulated that strategies that inhibit Th17 cell differentiation and promote Treg cell generation would be anticipated to suppress disease progression.

JAK-STAT signalling is an important pathway associated with autoimmune diseases such as SjD. Some studies have revealed a correlation between the JAK-STAT signalling pathway and SjD in the pathogenesis of SjD [10,11]. In our previous bioinformatics analysis, STAT1 was identified as one of the hub genes in SjD [12]. Several studies have revealed that overexpression of JAK and STAT family members is associated with the pathogenesis of SjD. In particular, STAT1, STAT3, and STAT5 have been identified as contributors to disease development, either individually or synergistically [13]. Suppression of JAK-STAT signaling can reduces IFN-γ induced CXCL10 production and attenuate immune-cell chemotaxis [14].

Tofacitinib (CP-690550) is a selective inhibitor of the JAK family with nanomolar potency and a high degree of kinome selectivity. It has been demonstrated to potently inhibit both JAK3-, JAK2- and JAK1-dependent STAT activation and γc cytokine signalling [15]. Currently, tofacitinib is clinically indicated for a range of autoimmune diseases such as ulcerative colitis, psoriatic arthritis, and ankylosing spondylitis [16–18]. *Ex vivo* studies have demonstrated that SjD patients exhibit elevated basal pSTAT levels in the minor salivary glands and peripheral blood mononuclear cells, which were corrected with tofacitinib [19]. However, there is a paucity of studies on the *in vivo* treatment of SjD with tofacitinib.

Therefore, we analyzed SjD-related transcriptomic arrays and hypothesized that tofacitinib, a JAK inhibitor, improves SjD-like features in murine models. In addition, we hypothesized that tofacitinib may alleviate secretory dysfunction by modulating Th17/Treg balance.

## Materials and methods

### Transcriptome sequencing analysis

We searched for SjD transcriptome sequencing data from the GEO database. Specifically, we identified salivary gland datasets (GSE159574 and GSE247662) [20,21], and listed related information in Table 1. Differentially expressed genes (DEGs) were identified using the DESeq2 method. Criteria for gene selection included a fold change of >1.2 and FDR of <0.05 [22]. The intersection of DEGs from GSE159574 and GSE247662 was obtained to identify common DEGs in salivary glands of human and murine SjD. These common DEGs were submitted to the STRING database with the interaction score set to 0.4. The top 10 genes were generated using the degree topological algorithm in Cytoscape. GO and KEGG pathway enrichment analysis were performed using the common DEGs. The drug target genes of interest were obtained by taking the intersection of the common DEGs and the dataset Cytokine-JAK-STAT signaling pathway (N00053) from the KEGG_MEDICUS of MsigDB database. The genes obtained from the intersection were uploaded to the DGIdb database to identify potential therapeutic drugs (interaction score > 0.8). Immune infiltration analysis was conducted using the Xcell method on the human data matrix (GSE159574). Immunohistochemistry (IHC) was used to validate the specific differential cells.

**Table 1.** Characteristics of the involved transcriptome sequencing datasets.

GSE ID	BioProject	Type	Control	Disease	Sample	PMID	Year
GSE159574	PRJNA669766	RNA-seq	13	16	Human salivary gland	33344450	2020
GSE247662	PRJNA1040125	RNA-seq	5	5	Murine submandibular gland	40545318	2023

### Animal grouping and drug administration

NOD/ShiLtJ mice are a widely used SjD animal model, whose symptoms include salivary and lacrimal gland lymphocyte infiltration; decreased salivary and lacrimal secretion; and positive anti-SSA, anti-SSB, anti-M3R, and ANA antibodies. The SjD-like autoimmune disorders appear in most female NOD/ShiLtJ mice at age 7–8 weeks and gradually develop. Therefore, mice older than 7 weeks were included. NOD/ShiLtj mice (female, aged 7 weeks) were purchased from Suzhou Cavens Biogle Model Animal Research Co. Ltd. Wild-type ICR mice (female, aged 7 weeks old) were purchased from Shanghai SLAC Laboratory Animal Co., Ltd., and were used as controls. All mice were bred in a specific pathogen-free barrier system at the Laboratory Animal Center of Fujian Medical University. The temperature of the feeding environment is 23 ± 2 °C and the humidity is 50% ∼ 60% with proper ventilation. Animals are adaptively fed one week before the experiment. At a baseline age of 8 weeks, mice were randomly caged, ear-tagged, and numbered HC1-24 (healthy control, ICR mice), V1-24 (vehicle, NOD/ShiLtj mice) and T1-24 (tofacitinib, NOD/ShiLtj mice). Random numbers were generated using the standard = RAND() function in Microsoft Excel. HC1-24 and V1-24 received daily intragastric administration with PEG300 (Abmole Bioscience, Houston, TX, USA). T1-24 was treated daily with tofacitinib (Abmole Bioscience, Houston, TX, USA) dissolved in PEG300 at a dose of 30 mg/kg/day administered orally. All mice were dosed with 0.2 ml/10 g once daily. The doses were administered for 0, 4, 8, and 12 weeks. At each time point, the mice in one group (*n* = 6) were sacrificed for further study. Mice are euthanized by intraperitoneal injection of overdose tribromoethanol before sample harvesting. Six mice in one group served as one experimental unit following a previous study [1]. Four different investigators were involved in the treatment, *in vivo* secretion-related assays, *ex vivo* assays, and formal analysis. The four investigators were blind to each other’s specific procedures and parameters.

### Fluorescein ocular surface staining

Mice were intraperitoneally anesthetized with 400 μL of 1.25% tribromoethanol (Aibei Biotechnology, Nanjing, JS, China) (*n* = 6). One microliter of a 1% sodium fluorescein solution (JINGMING, Tianjin, China) was instilled into the conjunctival sac. Transient eye movements were performed. Ocular surface staining was examined and imaged under cobalt blue light using a slit-lamp microscope (LS-4, Sunkingdom, Chongqing, China), and tear breakup time was recorded. Corneas were divided into four quadrants (superior temporal, inferior temporal, inferior nasal, and superior nasal), and each quadrant was scored 0–3 based on residual sodium fluorescein. The sum of the four quadrants was used as the staining score. The time from the first dry spot to tear break-up was recorded as tear break-up time.

### Tear flow rate

Each mouse received 400 μL of 1.25% tribromoethanol and 100 μL of 1% pilocarpine saline (Abmole Bioscience, Houston, TX, USA) intraperitoneally (*n* = 6). The reflexed end of phenol red cotton thread (JINGMING, Tianjin, China) was placed on the outer canthus of the eye and removed after 1 min. The length of the reddened part of the phenol red cotton thread was measured in millimeters. The tear flow rate was calculated using the formula: (reddened thread length/time/weight) × 100%.

### Histopathological assessment

The salivary glands were fixed in formalin, dehydrated in graded ethanol, and embedded in paraffin. Embedded tissues were sectioned at 4 μm, deparaffinized in xylene, and rehydrated in graded ethanol. The sections were stained with HE (Beyotime, Shanghai, China), AB-PAS (Solarbio, Beijing, China), Masson’s (Solarbio, Beijing, China), and Sirius red (Phygene, Fuzhou, Fujian, China) (*n* = 6). The histopathological criteria of the submandibular glands stained with HE were as follows: no acinar damage or lymphocytic infiltration was scored 0; mild acinar damage or mild lymphocytic infiltration was scored 1; moderate acinar damage or moderate lymphocytic infiltration was scored 2; moderate acinar damage or one lymphocytic infiltration focus was scored 3; multiple lymphocytic infiltration foci was scored 4.

### Saliva flow rate

Each mouse was administered 400 μL of 1.25% tribromoethanol intraperitoneally, in conjunction with 100 μL of 1% pilocarpine saline (*n* = 6). Cotton balls weighed before and after were placed at the submandibular duct opening for 10 min, and salivary flow was calculated using the formula: (saliva weight/time/body weight) × 100%.

### Immunohistochemistry

Salivary gland and spleen sections were deparaffinized and hydrated (*n* = 6). Antigen unmasking was performed by immersion in 1× citrate-EDTA heated to 120 °C for 15 min. After 30 min on the table, they were rinsed three times for 5 min each in ddH_2_O on a shaker table. Endogenous peroxidase activity was blocked with 3% hydrogen peroxide for 10 min. The primary antibodies used were all rabbit anti-mouse antibodies, including JAK3 (WL05400, 1:200, Wanlei Bio, Shenyang, China), phospho-JAK3 (340809, 1:200, ZenBio, Chengdu, China), STAT1 (WL02273, 1:200, Wanlei Bio, Shenyang, China), phospho-STAT1 (R25797, 1:200, ZenBio, Chengdu, China), CD4 (HA722966, 1:1000, HuaBio, Zhejiang, China), IL-17 (ER1902-37, 1:400, HuaBio, Zhejiang, China), RORA (HA723267, 1:1000, HuaBio, Zhejiang, China), CD25 (HA601486, 1:1000, HuaBio, Zhejiang, China) and FOXP3 (HA722835, 1:1000, HuaBio, Zhejiang, China). The slides were incubated overnight at 4 °C, washed with PBS, and incubated with biotin-labeled goat anti-rabbit IgG polymer (MXB, Fuzhou, Fujian, China) for 10 min at room temperature. Slides were thoroughly washed with PBS and incubated with streptavidin-peroxidase (MXB, Fuzhou, Fujian, China) for 10 min at room temperature. For optimal staining intensity, a freshly prepared DAB solution was applied to each section. A microscope (Axio Imager M2; Zeiss, Germany) was used to image the samples. Images were analyzed using the ImageJ (National Institutes of Health, Maryland, USA) plugin IHC Profiler, with the following scoring criteria: high positive = 4 points, positive = 3 points, low positive = 2 points, and negative = 1 point.

### Immunofluorescence

Mouse salivary gland tissues were fixed in 4% paraformaldehyde for 24 h, paraffin-embedded, and sectioned at 4 μm. After deparaffinization and rehydration, antigen retrieval was performed in citrate buffer (pH 6.0) at 95 °C for 20 min. Sections were blocked with 5% BSA for 1 h at 37 °C, then incubated with primary antibodies overnight at 4 °C, including CD4 (HA722966, 1:1000, HuaBio, Zhejiang, China), IL-17 (ER1902-37, 1:400, HuaBio, Zhejiang, China) and FOXP3 (HA722835, 1:1000, HuaBio, Zhejiang, China). After washing, sections were incubated with fluorophore-conjugated secondary antibodies for 1 h at 37 °C, followed by DAPI staining for 5 min. Images were captured using a microscope (Axio Imager M2; Zeiss, Germany).

### RT-qPCR

Total RNA was isolated from mouse submandibular glands using TRIzol reagent following the manufacturer’s instructions. RNA purity and concentration were determined by UV spectroscopy. Genomic DNA was removed using gDNA Erase at 42 °C for 2 min, and cDNA was then synthesized from 1 μg of total RNA. qPCR was performed in triplicate using SYBR Premix Ex Taq^™^ II (TaKaRa, Kyoto, Japan) on a Roche 480 Light Cycler (Roche, Mannheim, Germany). The cycling conditions were as follows: initial denaturation at 95 °C for 30 s, followed by 40 cycles of denaturation at 95 °C for 5 s and annealing/extension at 60 °C for 30 s. With *Gapdh* as the internal control, the relative expression levels of target genes were calculated using the 2^−ΔΔCt^ method. Primer sequences are listed below: *Gapdh* (F: 5′-ACAACTTTGGCATTGTGGAA-3′, R: 5′-GATGCAGGGATGATGTTCTG-3′); *Il-17a* (F: 5′-GCTCCAGAAGGCCCTCAGA-3′, R: 5′-AGCTTTCCCTCCGCATTGA-3′); *Foxp3* (F: 5′-CACCTATGCCACCCTTATCGG-3′, R: 5′-CATGCGAGTAAACCAATGGTA-3′).

### Flow cytometry

Naive T cells in splenocytes were activated with plate-bound anti-CD3 antibody (5 mg/ml), anti-CD28 antibody (2 mg/ml), and ConA (3 μg/ml) (Multisciences, Hangzhou, Zhejiang, China) in OptiVitro T cell-serum-free medium (ExCell, Taicang, Jiangsu, China) for 3 days (*n* = 3). Single-cell suspensions were prepared in OptiVitro T cell serum-free medium at a density of 1 × 10^6^ cells/ml. Cells were seeded into 24-well plates and incubated with PMA/ionomycin and a BFA/monensin mixture (250×) (Multisciences, Hangzhou, Zhejiang, China) at 37 °C for 6 h. The cells were then stained with fluorescence-conjugated antibodies against CD3, CD4, CD25, and FOXP3 (Multisciences, Hangzhou, Zhejiang, China), and intracellular staining was performed according to the manufacturer’s recommendations. Samples were analyzed using FCM (Accuri C6 Plus, BD, USA), and data were analyzed using Flowjo 7.6 software (Treestar, USA).

### Cytokine measurement

The plasma concentrations of IL-17, IL-10, and TGF-β were measured using a commercially available ELISA kit (Alpha Diagnostic International, San Antonio, TX, USA; ABclonal, Wuhan, Hubei, China). Each plasma sample was run in duplicate.10 μL of plasma (diluted 1:10) were added to the ELISA plates to measure the levels of autoantibodies and cytokines, following the manufacturer’s instructions (*n* = 5 or 6). The absorbance of the entire plate was read at 450 nm using a spectrophotometer (SpectraMax iD3, Molecular Devices, San Jose, CA, USA) within 30 min of addition of the termination solution. Absorbance at 630 nm was measured to normalize the background.

### Statistical analysis

All data are expressed as mean ± SD. Statistical analysis was performed using the GraphPad Prism 8.0 software package (GraphPad Software Inc., San Diego, CA, USA). Normality and homogeneity of variance of the data were checked using the Shapiro-Wilk test and Leven’s test. Results were analyzed by ANOVA and multiple comparisons between groups by the Tukey test, and associations between two continuous variables were analyzed by Pearson correlation analysis. A *p* significance was set at *p* < 0.05.

## Results

### JAK-STAT pathway and CD4^+^ T cells were involved in SjD onset while tofacitinib acted as a therapeutic agent

**Figure 1.**
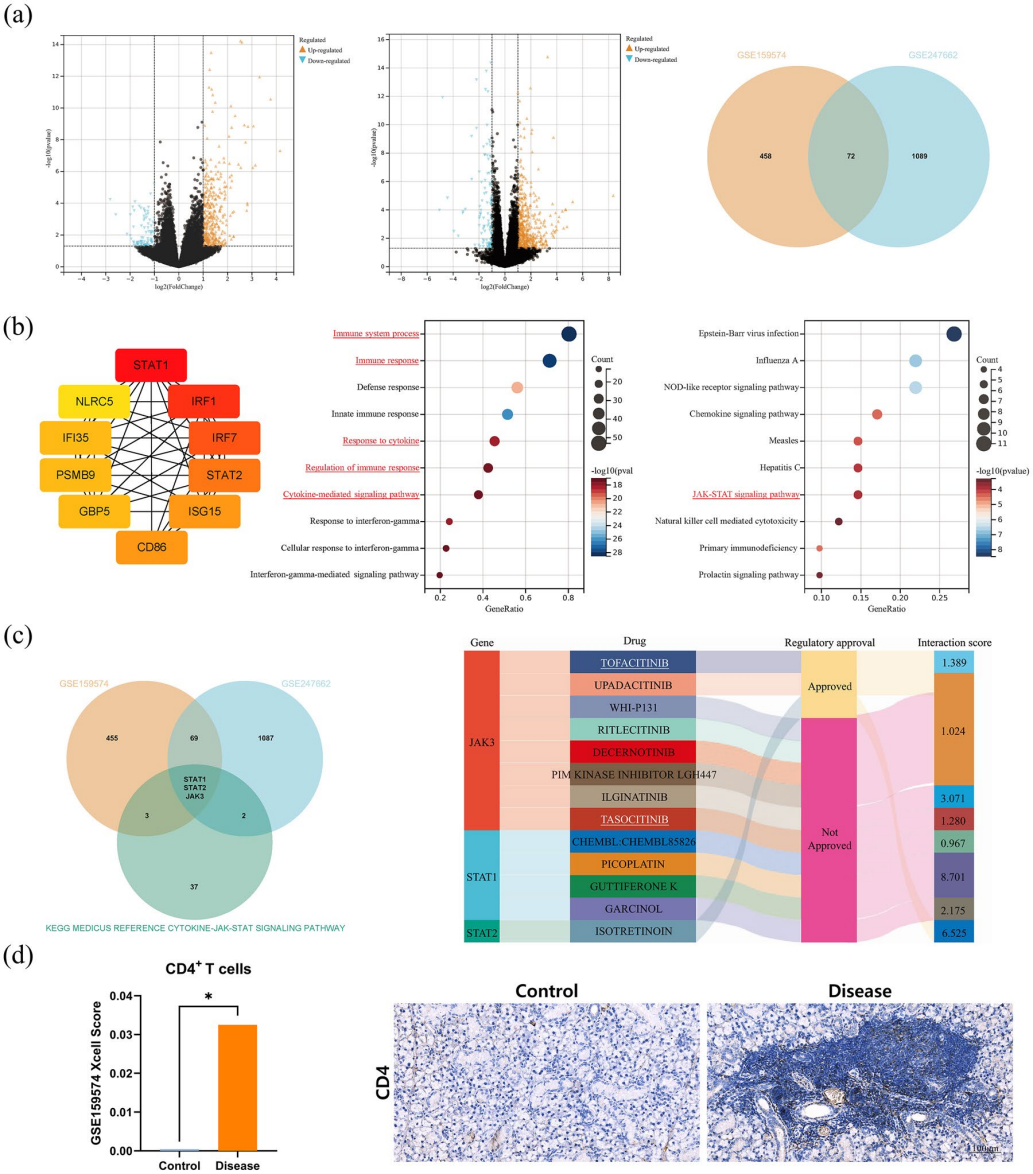
Integrated analysis of human and murine transcriptome Sequencing in SjD. (a) Profiles of GSE159574 and GSE247662 and their common DEGs; (b) Top10 DEGs in the PPI network, GO and KEGG enrichment analysis of common DEGs; (c) Intersection of common DEGs and cytokine-JAK-STAT pathway genes, and their associated potential therapeutic agents; (d) Xcell analysis of CD4^+^ T cells and IHC validation, *p < 0.05.

The GSE159574 and GSE247662 datasets were confirmed to contain 530 and 1161 DEGs respectively, with 72 common DEGs identified between them (Figure 1(a)). Analysis of these 72 DEGs revealed the following: PPI network analysis screened out the top 10 hub genes with STAT1 as the core; GO analysis was mainly enriched in terms related to immune response and cytokines; KEGG analysis suggested that the JAK-STAT pathway was involved in the pathogenesis of SjD (Figure 1(b)). After intersecting these 72 DEGs with genes of Cytokine-JAK-STAT signaling pathway in the KEGG_MEDICUS database, a total of 3 drug target genes were obtained: JAK3, STAT1, and STAT2. Results from the DIGdb database indicated that tofacitinib was a potential therapeutic agent for SjD (Figure 1(c)). Furthermore, Xcell analysis showed that the infiltration level of CD4^+^ T cells in SjD patients was higher than that in the control group, and this result was further validated by salivary gland IHC in mouse models (Figure 1(d)).

### Tofacitinib alleviated tear secretory dysfunction

**Figure 2.**
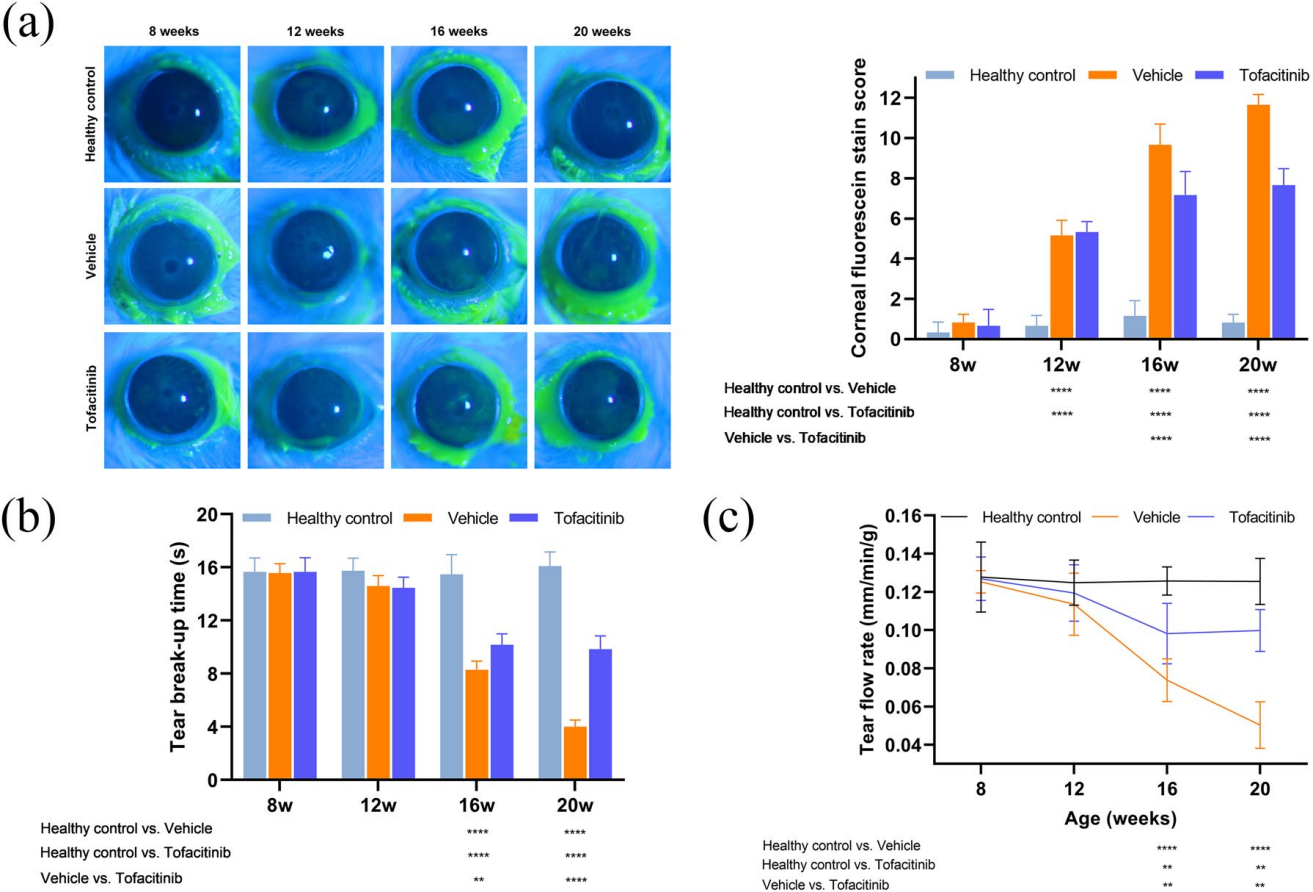
Tofacitinib reduced the corneal fluorescein staining score, prolonged the tear break-up time, and improved the tear flow rate. (a) Corneal fluorescein staining score. (b) Tear break-up time. (c) Tear flow rate. *n* = 6, **p < 0.01, ***p < 0.005, ^****^p < 0.001.

After identifying pathogenesis-related immune cells and pathways, we selected a modulatory medicine, the JAK inhibitor tofacitinib. To evaluate the effect of tofacitinib on sicca symptoms, the tear secretory function of the SjD models (NOD/ShiLtj mice) was observed. The positive staining area of corneal fluorescein in the vehicle and tofacitinib groups gradually increased from homogeneous to punctate, patchy, and lamellar. Tofacitinib significantly decreased corneal fluorescein staining at weeks 16 and 20 (Figure 2(a)). Similarly, at weeks 16 and 20, tofacitinib prolonged tear breakup time (Figure 2(b)) and tear flow was higher in the tofacitinib group than in the vehicle group (Figure 2(c)). Overall, tofacitinib improved tear secretory function in NOD/ShiLtj mice.

### Tofacitinib alleviated autoimmune sialadenitis histologically and decreased phospho-JAK3-STAT1 expression

**Figure 3.**
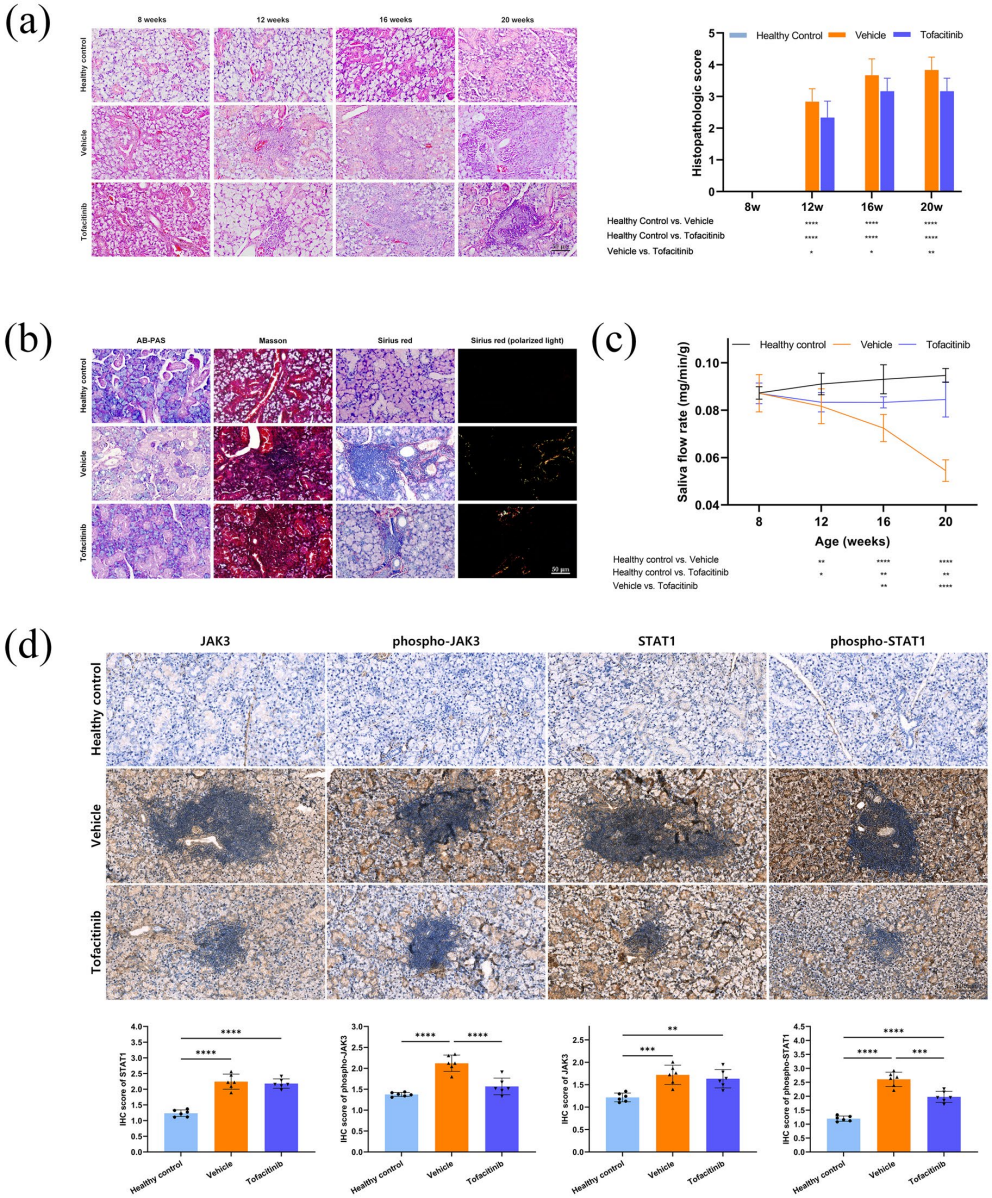
Tofacitinib reduced lymphocyte infiltration, changed structures, improved saliva flow rate, and decreased phospho-JAK3-STAT1 levels of salivary glands. (a) HE staining (400×, bar = 50 μm) and histopathologic score, *n* = 6. (b) AB-PAS, Masson, Sirius red staining of week 20 mice’s submandibular glands (400×, bar = 50 μm). (c) Saliva flow rate, *n* = 6. (d) Expression of JAK3, phospho-JAK3, STAT1, phospho-STAT1 (400×, bar = 100 μm) and immunohistochemistry score, *n* = 6. *p < 0.05, **p < 0.01, ***p < 0.005,^****^p < 0.001.

Histopathological assessment was used to investigate the effect of tofacitinib on local inflammation in the submandibular glands. As the disease progressed, lymphocytic infiltration gradually increased in both vehicle and tofacitinib groups. After 4 weeks of tofacitinib administration, lymphocyte infiltration in NOD/ShiLtj mice significantly improved and acinar damage was reduced (Figure 3(a)). AB-PAS staining revealed higher mucinogen, acidic mucopolysaccharide, and neutral mucopolysaccharide levels in the tofacitinib group than in the vehicle group. Masson’s staining showed a decrease in the collagen-positive blue area surrounding lymphocytic infiltrates in the tofacitinib group compared to the vehicle group. Under polarized light using Sirius Red staining, the vehicle group had more green type III collagen fibers and the tofacitinib group had more orange type I collagen fibers. The structure of salivary glands was altered by tofacitinib treatment (Figure 3(b)). After 8 weeks of oral tofacitinib treatment, the salivary flow rate was higher in the tofacitinib group than in the vehicle group (Figure 3(c)). There was also a significant reduction in the nuclear and cytoplasmic staining levels of phospho-JAK3 and phospho-STAT1 in the tofacitinib group compared to those in the vehicle group. Meanwhile, the expression levels of JAK3 and STAT1 showed no significant difference. This indicated that tofacitinib exerted its effects primarily by inhibiting the phosphorylation of JAK3 and STAT1 in the submandibular glands (Figure 3(d)).

### Tofacitinib inhibited Th17 response but promoted Treg response in SjD salivary glands

**Figure 4.**
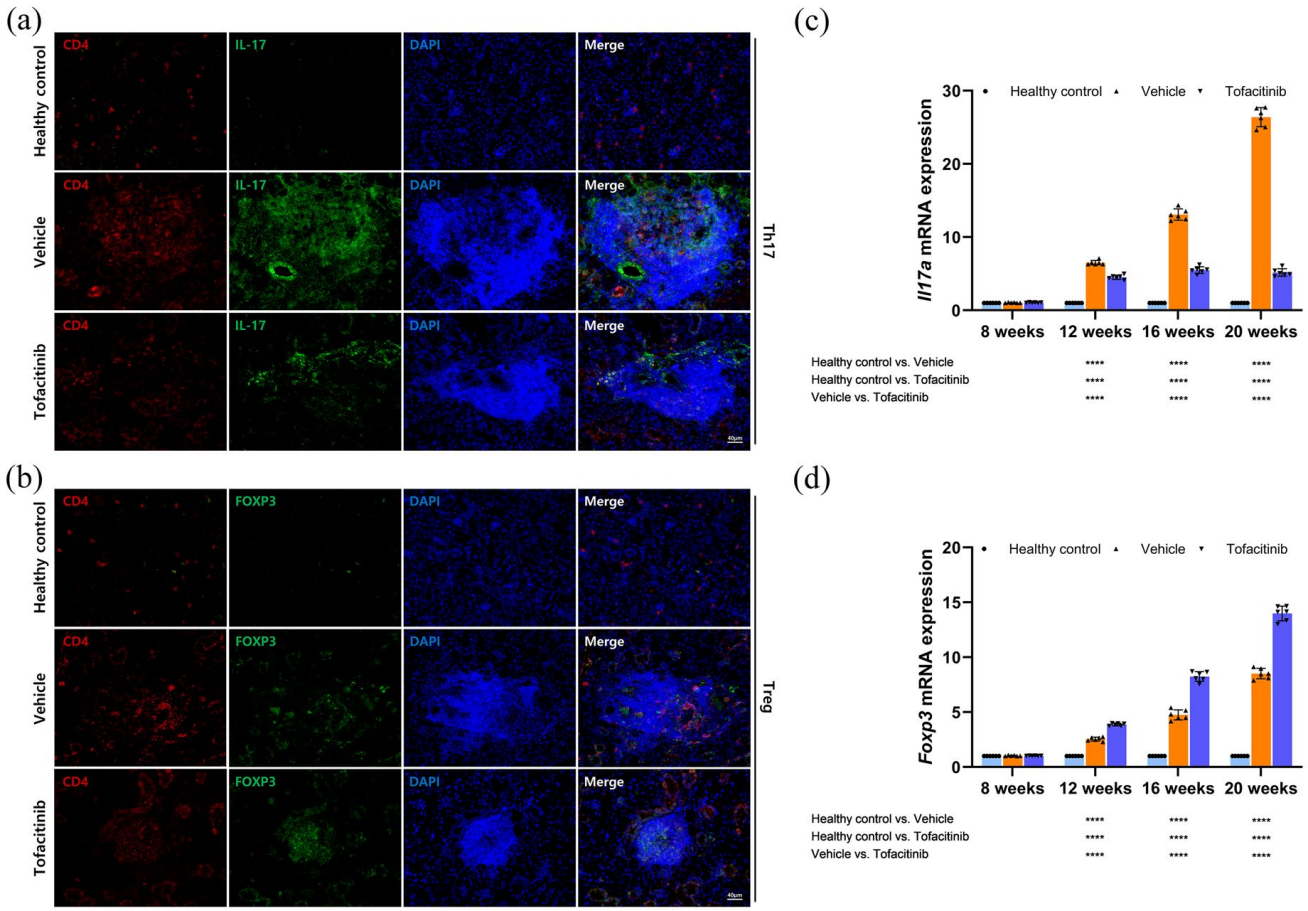
Tofacitinib diminished Th17 cell infiltration in SjD-associated salivary glands, with a concurrent increase in Treg cell infiltration. (a) Immunofluorescence of CD4^+^IL-17^+^ cells in submandibular glands of 20-week-old murine models. (b) Immunofluorescence of CD4^+^FOXP3^+^ cells in submandibular glands of 20-week-old murine models. (c) *Il17a* mRNA expression levels in murine submandibular glands. (d) *Foxp3* mRNA expression levels in murine submandibular glands.

In submandibular gland tissues of 20-week-old SjD murine models, CD4^+^ cells were labeled with Cy3 (red fluorescence), while IL-17 and FOXP3 were labeled with Alexa Fluor 488 (green fluorescence). Colocalization signals of CD4 with IL-17 and of CD4 with FOXP3 were observed in lymphocytic infiltrates. The abundance of CD4^+^IL-17^+^ cells in lymphocytic infiltrates was higher in the vehicle group than in the tofacitinib group (Figure 4(a)), whereas the abundance of CD4^+^FOXP3^+^ cells was higher in the tofacitinib group than in the vehicle group (Figure 4(b)). RT-qPCR results also showed that the expression of *Il17a* was consistently higher in the vehicle group than in the tofacitinib group at 12, 16, and 20 weeks (Figure 4(c)), while the expression of *Foxp3* was consistently higher in the tofacitinib group than in the vehicle group (Figure 4(d)). These results suggested that tofacitinib reduced Th17 cell (CD4^+^IL-17A^+^) responses while promoting Treg cell (CD4^+^Foxp3^+^) responses in the submandibular glands of SjD murine models.

### Tofacitinib suppressed Th17 responses while enhancing Treg responses in SjD spleens

**Figure 5.**
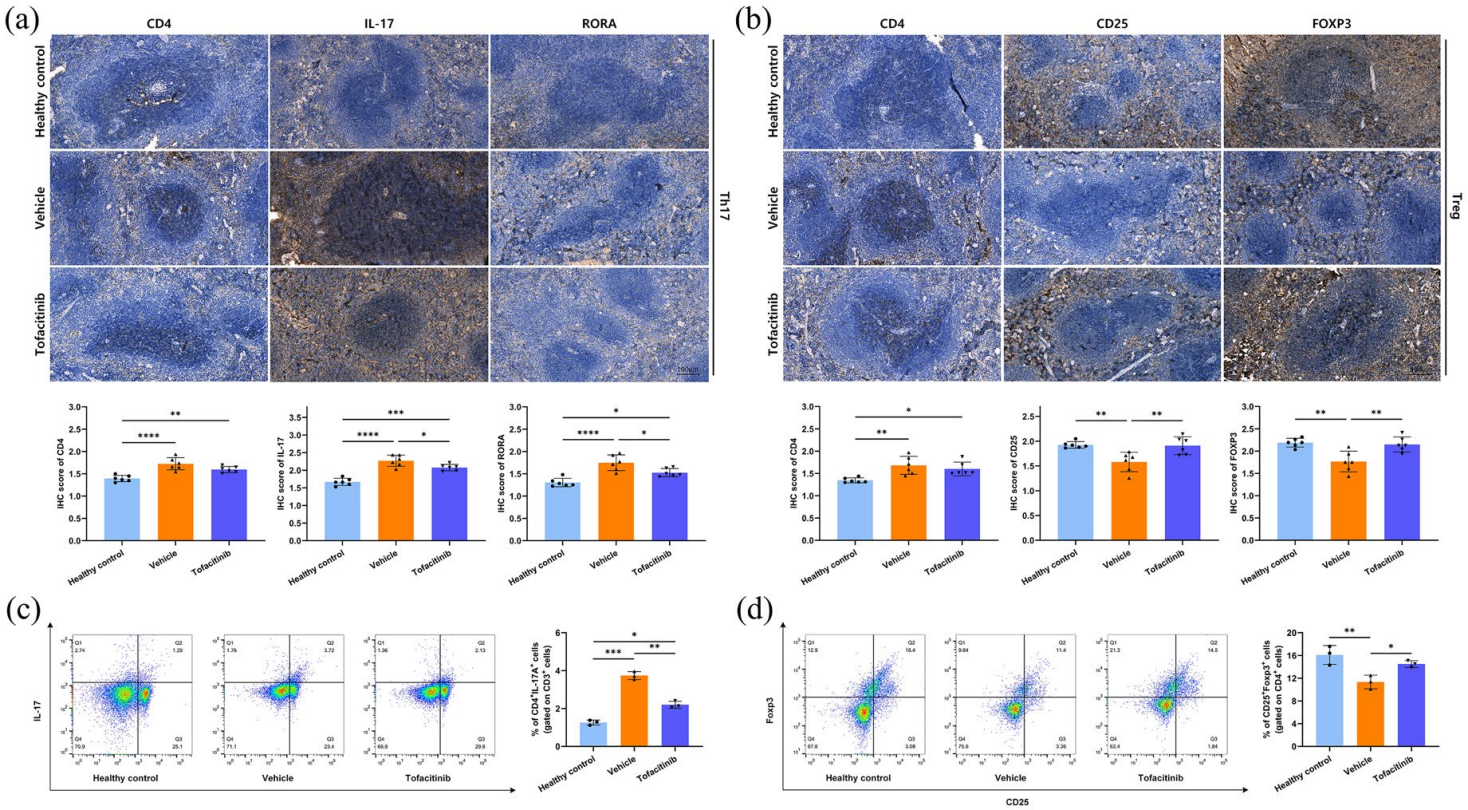
Tofacitinib suppressed Th17 cell responses while concurrently enhancing Treg cell responses in the spleens of 20-week-old SjD murine model. (a) Immunohistochemistry of CD4^+^, IL-17^+^, and RORA^+^ cells in spleens. (b) Immunohisto­chemistry of CD4^+^, CD25^+^, and FOXP3^+^ cells in spleen. (c) Th17 proportion in spleens, *n* = 3. (d) Treg proportion in spleens, *n* = 3. *p < 0.05, **p < 0.01, ***p < 0.005, ^****^p < 0.001.

IHC results showed that in 20-week-old murine models, the numbers of splenic CD4^+^ cells in the healthy control group were generally lower than those in the vehicle group and the tofacitinib group. Meanwhile, the numbers of IL-17^+^ and RORA^+^ cells in the vehicle group were higher than those in the tofacitinib group (Figure 5 (a)), while the numbers of CD25^+^ and FOXP3^+^ cells in the tofacitinib group were higher than those in the vehicle group (Figure 5 (b)). The proportion of Th17 cells among splenic CD3^+^ T cells was lower in the tofacitinib group than in the vehicle group, indicating a reduction in Th17 (CD4^+^IL-17^+^) cell subsets after 12 weeks of tofacitinib treatment (Figure 5 (c)). The proportion of Treg (CD25^+^Foxp3^+^) cell subsets was higher in the tofacitinib group than in the vehicle group, suggesting that 12 weeks of tofacitinib treatment may increase the proportion of Treg cells among splenic CD4^+^ T cells (Figure 5(d)). These results suggested that tofacitinib ameliorated the Th17/Treg cell imbalance in the spleen of SjD murine models.

### Tofacitinib downregulated Th17-related cytokines and concurrently upregulated Treg-related cytokines in SjD plasma

**Figure 6.**
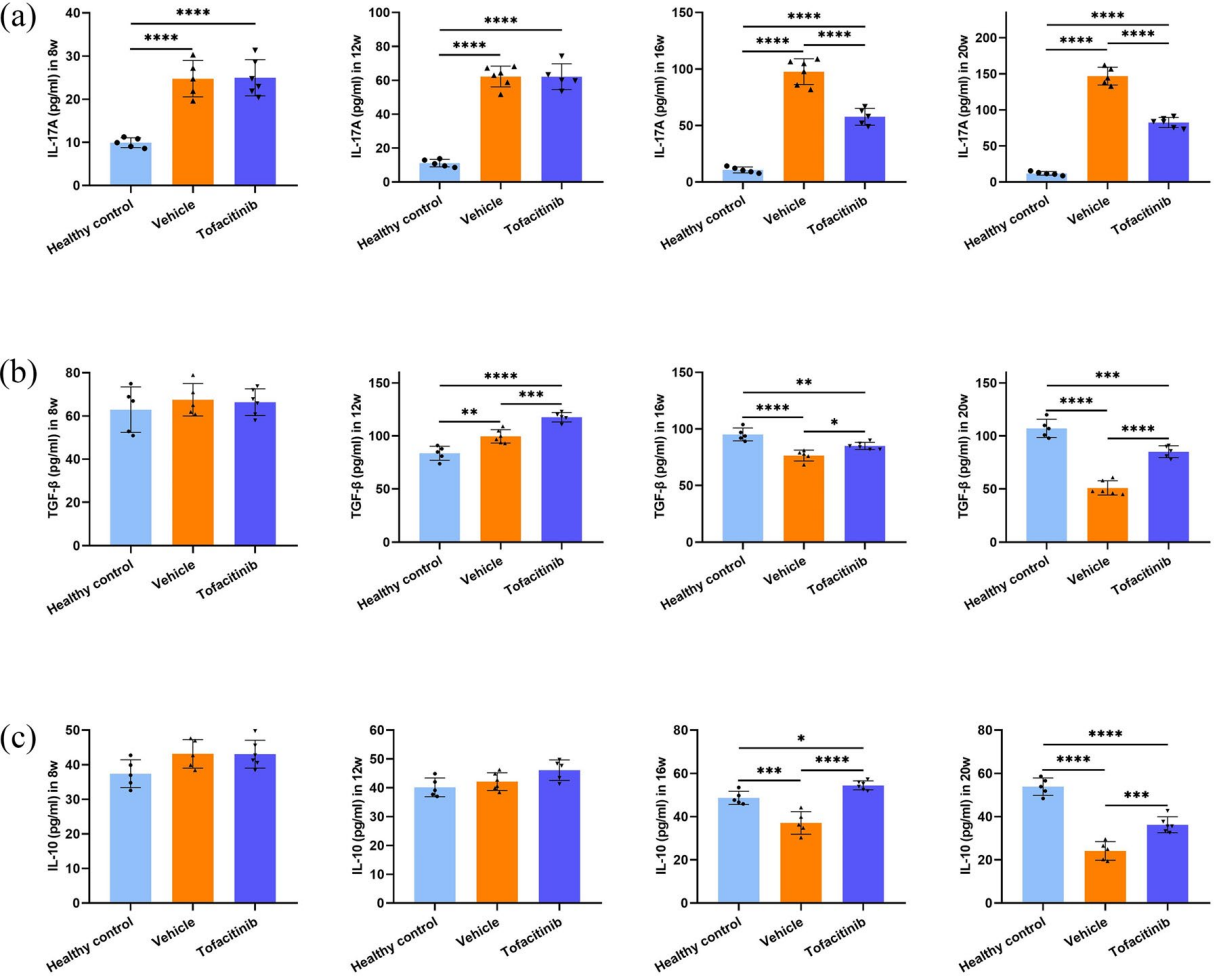
Tofacitinib inhibited Th17 response but promoted Treg response. (a) IL-17 concentration in plasma, *n* = 5/6. (b) IL-10 concentration in plasma, *n* = 5/6. (c) TGF-β concentration in plasma, *n* = 5/6. *p < 0.05, **p < 0.01, ***p < 0.005, ^****^p < 0.001.

ELISA of plasma showed that IL-17A levels were lower in the tofacitinib group than in the vehicle group at weeks 16 and 20, with tofacitinib gradually reducing plasma IL-17A levels to near normal levels (Figure 6(a)). In addition, at weeks 16 and 20, the levels of IL-10 (Figure 6(b)) and TGF-β (Figure 6(c)) were higher in the tofacitinib group than in the vehicle group, suggesting that tofacitinib could increase the plasma concentrations of IL-10 and TGF-β. IL-17A is secreted by Th17 cells, whereas IL-10 and TGF-β are secreted by Tregs. Plasma cytokines indicate the abundance of T cell subsets in peripheral blood. Therefore, tofacitinib decreased the proportion of Th17 cells but increased the number of Treg cells in the peripheral blood.

### Th17/Treg balance was highly related to the secretory function

**Figure 7.**
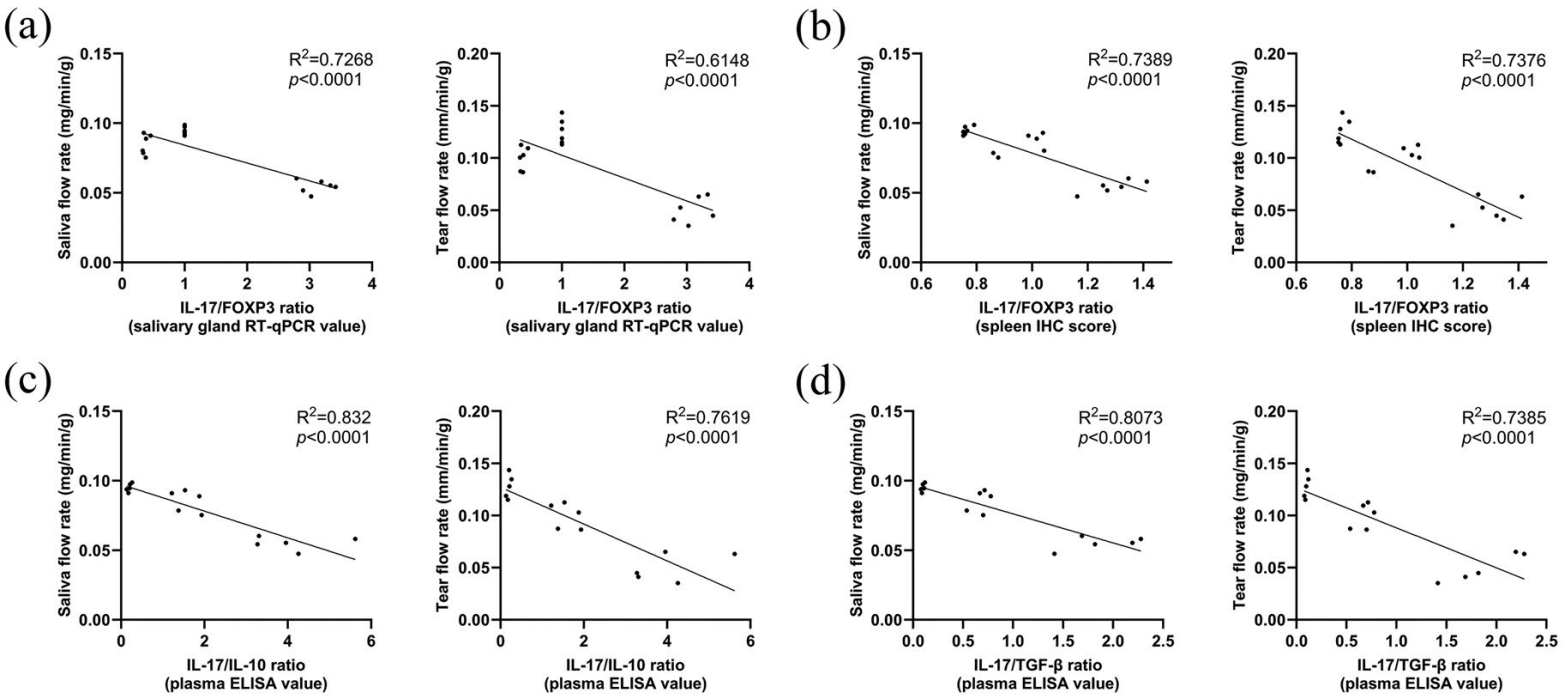
IL-17/FOXP3, IL-17/IL-10, and IL-17/TGF-β ratio were highly related to saliva and tear flow rate. (b) Pearson’s linear regression between IL-17/FOXP3 ratio (salivary gland RT-qPCR value) and saliva/tear flow rate, *n* = 18. (b) Pearson’s linear regression between IL-17/FOXP3 ratio (spleen IHC score) and saliva/tear flow rate, *n* = 18. (c) Pearson’s linear regression between IL-17/IL-10 ratio (plasma ELISA value) and saliva/tear flow rate, *n* = 15. (d) Pearson’s linear regression between IL-17/TGF-β ratio (plasma ELISA value) and saliva/tear flow rate, *n* = 15.

Pearson’s linear regression was utilized to examine the correlation between the Th17/Treg balance and secretory function. The ratios of IL-17/FOXP3, IL-17/IL-10, and IL-17/TGF-β were found to be associated with saliva/tear flow rates. The IL-17/FOXP3 ratio (salivary gland RT-qPCR value) was also found to have a strong negative correlation with saliva/tear flow rates (R^2^ = 0.7268/0.6148, *p* < 0.0001) (Figure 7(a)). The IL-17/FOXP3 ratio (spleen IHC score) was also found to have a strong negative correlation with saliva/tear flow rates (R^2^ = 0.7389/0.7376, *p* < 0.0001) (Figure 7(b)). Similarly, the Pearson correlation analysis between the IL-17/IL-10 ratio (plasma ELISA value) and saliva/tear flow rates revealed a strong negative relationship (R^2^ = 0.832/0.7619, *p* < 0.0001) (Figure 7(c)). The Pearson correlation analysis showed a strong negative correlation between the IL-17/TGF-β ratio (plasma ELISA value) and saliva/tear flow rates (R^2^ = 0.8073/0.7385, *p* < 0.0001) (Figure 7(d)). These findings suggest that the IL-17/FOXP3, IL-17/IL-10, and IL-17/TGF-β ratios could serve as a marker for the Th17/Treg balance in SjD due to its strong association with secretory function.

## Discussion

In the current study, We integrated the human SjD dataset GSE159574 and the NOD/ShiLtj murine dataset GSE247662. Analysis of common DEGs revealed that the JAK-STAT pathway and CD4^+^ T cells played roles in SjD pathogenesis, while tofacitinib was a potential therapeutic agent for SjD. By establishing a tofacitinib-administered model using NOD/ShiLtj mice, we found that inhibition of the JAK-STAT pathway could effectively alleviate the secretory dysfunction symptoms of SjD and reduce tissue inflammatory infiltration by regulating the Th17/Treg balance.

SjD is recognized as a complex disease with a multigene host background that determines the immune cell phenotype and disease progression [23]. Autoimmunity, immune dysregulation, and epithelial dysfunction are the underlying pathophysiological mechanisms [2]. Therefore, it is important to identify the susceptible immune cells and gene pathways of SjD to study the cause of this disease and to identify potential treatments. Two RNA-sequencing datasets were employed in this study. Both the human SjD dataset GSE159574 and NOD/ShiLtj murine dataset GSE247662 contained salivary gland samples, which were affected organs in autoimmunity. Given the limited sample size and to prevent missing functional regulatory genes (e.g. transcription factors, cytokine receptors), we used FC > 1.2 for DEGs screening, which filters most irrelevant noise. FC > 1.2 is well-suited for conventional experiments with moderate sample sizes and data variability. Moreover, to focus on strongly DEGs (e.g. direct drug targets), we further added FDR < 0.05 as a criterion. The enrichment results of DEGs point to the JAK-STAT signalling pathway, which bridges extracellular inflammation-related cytokines and intracellular effector proteins [13]. JAKi, such as tofacitinib, have been widely studied in various mouse models to investigate their effects on autoinflammatory diseases by inhibiting JAK-STAT pathways. Furthermore, immune cell infiltration analysis of human SjD datasets revealed differences in CD4^+^ T cells between the disease group and the healthy control group. Th17 and Treg cells are specific functional subsets differentiated from CD4^+^T cells by distinct microenvironmental signals, with complementary yet mutually restrictive functions that jointly regulate immune homeostasis. Thus, this study aims to explain the immunological mechanisms underlying the therapeutic effects of tofacitinib on SjD through the Th17/Treg balance [24].

As a semi-selective JAK inhibitor (not a pan-JAKi), tofacitinib exerts its primary inhibitory effects on JAK1 and JAK3, which in turn impacts the downstream signaling of all homodimeric and heterodimeric JAK complexes. This mechanism of action is relevant across species, including humans and mice. Specifically in humans, JAK3 exhibits inducible expression and acts as a regulatory ‘rheostat’ to enhance the amplitude of cytokine. The administration of tofacitinib has been demonstrated to alleviate joint injury and inflammatory response in mice with collagen-induced arthritis (CIA). Tofacitinib was shown to restore the balance between γδTreg and γδTh17 cells in rheumatoid arthritis and to inhibit excessive NLRP3 inflammasome activation. Furthermore, tofacitinib was observed to inhibit the activation of γδTh17 cells *via* the suppression of the NLRP3 inflammasome [25]. Delayed and limited administration of tofacitinib mitigates chronic dextran sulfate sodium (DSS) induced colitis in C57BL/6J mice. Tofacitinib has an alleviating effect on inflammatory bowel disease. The ratio of CD4^+^ to CD8^+^ T cells increased as a consequence of tofacitinib treatment. Furthermore, the frequency of CD4^+^ regulatory T cells (Tregs) within CD4^+^ T-cell populations was elevated. Late administration of tofacitinib to colitis mice reduced the abundance of IFN-γ^+^ T cells and conversely enriched IL-10^+^ T cells [26]. In addition, tofacitinib treatment reduced serum levels of pro-inflammatory cytokines including TNF-α, IFN-γ, IL-2 and MIP1-α in MRL/lpr lupus-prone mice [27]. Other Jaki, such as baricitinib, have been demonstrated to ameliorate MOG35-55 and pertussis toxin-induced experimental autoimmune encephalomyelitis in C57BL/6J mice. The baricitinib therapeutic group exhibited a notable reduction in the proportion of CD4^+^IFN-γ^+^ Th1 and CD4^+^IL-17^+^ Th17 cells. The expression of cytokines IL-2, IL-6, IL-12, IL-17, IL-23, IFN-γ, and TNF-α was markedly diminished in the baricitinib treatment groups [28].

These results in this study indicate that tofacitinib exerts a suppressive effect on inflammatory responses and secretory phenotype deterioration as a JAK inhibitor in a multitude of tissues. Tofacitinib significantly attenuated dry eye symptoms in NOD/ShiLtJ mice. Dry eye can be attributed to immune-mediated inflammatory damage to the lacrimal gland due to high tear osmolarity and tear film instability [29]. Tear film instability also contributed to the premature tear film rupture, forming punctate and lamellar staining in corneal staining assay. Diminished exocrine gland function in SjD patients may be associated with antibodies to aquaporins, especially to AQP8 and AQP9 [30]. In the histopathological assays in this study, a reduction in the number of infiltrating lymphocytes in the interstitium was observed following tofacitinib administration. In addition, mucous acini are less damaged by inflammation, but the content of mucinogen, acidic mucopolysaccharide, and neutral mucopolysaccharide in mucous acini decreases significantly according to AB-PAS staining [31]. The increased amount of collagen near the foci of lymphocyte infiltration and the altered collagen type might be related to local tissue alterations caused by inflammation [32,33]. The activation of STAT1, STAT6, and STAT3 drives CD4^+^ T cell responses, including Th1, Th2, Th17, and Treg cells [34]. STAT1, STAT3, and STAT6 phosphorylation were inhibited in the submandibular glands treated with tofacitinib. Activation of STAT5 prompts a Treg response, producing local anti-inflammatory effects [35]. Higher STAT5 phosphorylation after tofacitinib treatment may be related to the negative feedback from downstream molecules.

It has been acknowledged that Th17 and Treg cells play important roles in the pathogenesis of various autoimmune diseases, including SjD. In comparison with ICR mice, the number of Th17 cells with CXCR3 was higher and the number of Treg cells with CXCR3 was lower in the spleens of NOD mice at 4 weeks without sicca symptoms [36]. Patients can often have a decrease in peripheral blood T cells in the early stages due to the gradual migration of T cells into the exocrine glands [37]. Th17-related cytokines are expressed at higher levels in the salivary glands of patients with SjD [8]. Th17 cells trigger autoimmunity, while Tregs expand in response to cytokine stimulation and suppress autoreactive cells [38]. Tofacitinib can affect the downstream proteins of JAK-STAT by inhibiting the JAK protein in T cells. The inhibition of CD4^+^ and CD8^+^ T cell proliferation, along with the suppression of Th17 differentiation, was observed in the presence of tofacitinib [39]. Tofacitinib can increase the frequency and effector phenotype of Tregs [25]. SHR0302, a JAK inhibitor, reduces Th17 function by inhibiting JAK1-STAT3 phosphorylation [40]. Tofacitinib treatment increased the Treg response and reduced the Th17 response in immune-mediated liver injury [41]. Tolerogenic dendritic cells generated with tofacitinib ameliorate experimental autoimmune encephalomyelitis by modulating Th17/Treg balance [42]. In this study, the proportion of CD3^+^CD4^+^IL-17^+^ T cells was lower in the treatment group than in the vehicle group, whereas the proportion of CD4^+^CD25^+^Foxp3^+^ T cells was higher in the treatment group than in the vehicle group. Plasma IL-17 levels decreased, while IL-10 and TGF-β levels increased in the tofacitinib-treated group. Namely, with increasing periods of tofacitinib administration, pro-inflammatory Th17 cells decreased while anti-inflammatory Tregs increased *in vivo*. The Th17/Treg balance was slowly restored and disease development was ameliorated.

Given that the differentiation of Th17 and Treg cells is reciprocally regulated by shared and different cytokines, and that each subset can convert to the other under certain inflammatory conditions, it is not surprising that the Th17/Treg balance plays a crucial role in the pathogenesis of SjD. In the present study, the Th17/Treg balance was represented as the IL-17/FOXP3 ratio in the spleen, IL-17/IL-10 ratio in plasma, and IL-17/TGF-β ratio in plasma, which jointly promote the onset of SjD. Similarly, tear and saliva secretions are directly related to gland damage. A strong negative correlation between the Th17/Treg balance and secretory function was observed in this study. This suggests that the ratios of Th17/Treg, IL-17/IL-10, and IL-17/TGF-β may serve as markers of Th17/Treg balance, providing a means of monitoring the secretory function of patients with SjD. In IL-17^-/-^ SjD murine models, there is a notable decrease in glandular histopathological damage and a marked enhancement in the functionality of salivary and lacrimal gland secretions [43]. An increased proportion of Treg cells was observed in conjunction with elevated levels of IL-10, leading to enhanced disease profiling of SjD [44]. Similar to tofacitinib, IL-2 treatment can reduce JAK3 phosphorylation [45]. Short-term low‐dose IL‐2 therapy can restore Th17/Treg balance in the blood of patients with SjD and control disease activity [24]. Therefore, it is important to focus on the Th17/Treg balance when addressing the therapeutic needs of patients with SjD.

Indeed, a limitation of our study is that we used female NOD/Ltj mice as models. NOD/Ltj mice developed type 1 diabetes during our treatment. Although we found that there was no correlation between blood sugar and saliva secretion, another model without type 1 diabetes or pancreatitis, like C57BL/6J.NOD-Aec1Aec2 may be more suitable [46].

## Conclusions

In summary, tofacitinib has the potential to enhance secretory function in mice with SjD and decrease inflammation levels. The therapeutic effect of the JAK inhibitor, tofacitinib, on SjD may be attributed to its ability to modulate the balance between Th17 and Treg cells.

## Supplementary Material

Supplemental Material

Gating.jpg

## Data Availability

The data that support the findings of this study are available from the corresponding author, JC, upon reasonable request.
