# Supplementary figures and images for: JAK inhibitor tofacitinib alleviates secretory dysfunction and Th17/Treg imbalance in a Sjögren’s disease murine model
Source: Ann Med. 2026 Feb 8;58(1):2625552. doi: 10.1080/07853890.2026.2625552 (PMC12888350; doi:10.1080/07853890.2026.2625552)

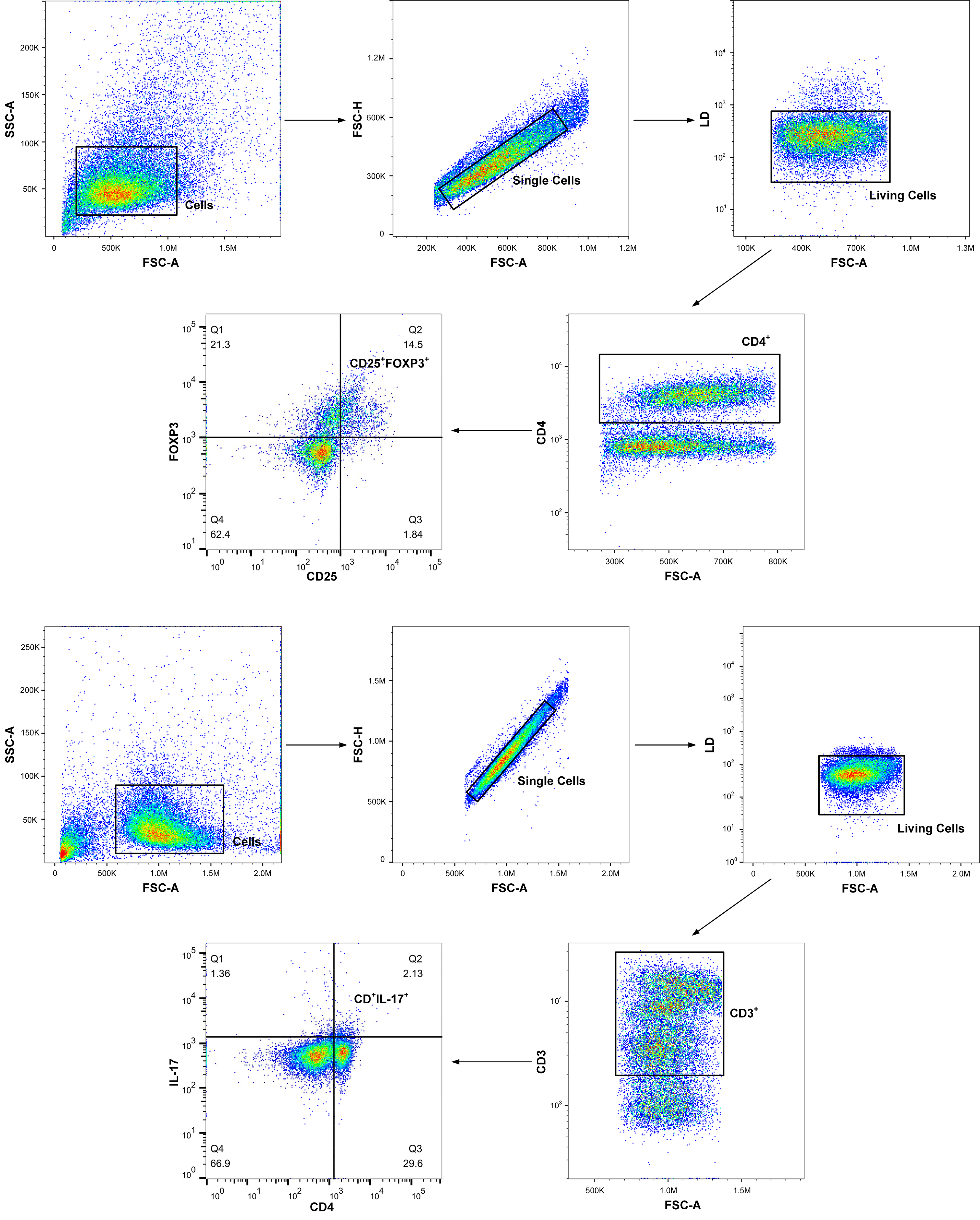

Supplement: Gating.jpg [file IANN_A_2625552_SM0274.jpg]
